# The perspective of manual therapy as a fascia-directed biomechanical intervention in myofascial pain syndrome

**DOI:** 10.3389/fmed.2026.1823862

**Published:** 2026-04-30

**Authors:** Haiteng Wang, Peizhen Zhang, Jianwu Wang, Junshi Wang, Junlong Li, Mingzhu Pan

**Affiliations:** 1 Tuina Department, National Clinical Research Center for Chinese Medicine Acupuncture and Moxibustion, First Teaching Hospital of Tianjin University of Traditional Chinese Medicine, Tianjin, China; 2 National Administration of Traditional Chinese Medicine Level Three Laboratory for Tuina Technique Biological Effects, Tianjin, China

**Keywords:** biomechanics, fascial network, manual therapy, myofascial pain syndrome, neuromodulation

## Introduction

Epidemiological studies indicate that myofascial pain syndrome (MPS) may affect up to 20% of the general population, with reported prevalence rates as high as 85% to 93% in pain specialist clinics (1, 2). Among patients with non-specific neck pain, the prevalence of MPS reported in different studies varies considerably, with some studies indicating a relatively high prevalence (3). This variation reflects inconsistencies in the diagnostic criteria for MPS and suggests that we should interpret prevalence data with caution.

Over 52,000 publications concerning MPS have been published on PubMed. Myofascial pain syndrome (MPS) involves musculoskeletal pain characterized by localized and radiating discomfort, perceived as dull aching, pressure, or soreness, alongside myofascial trigger points that may occur anywhere on the body. According to data from the National Institutes of Health (NIH), multiple funded projects over the past 2 years have had primary or secondary research focus on the diagnosis and treatment of MPS, including therapeutic approaches such as myofascial release (4). The muscular and neural components of MPS have been extensively studied, encompassing motor, sensory, and autonomic nervous system elements. However, the role of fascia within the MPS field remains under-researched; the term “myofascial” denotes the union of muscle and its surrounding connective tissue. Myofascial pain syndrome (MPS) is a recurrent or chronic musculoskeletal disorder characterized by localized pain and tenderness in specific areas of muscle and fascia, typically accompanied by hypersensitive nodules known as myofascial trigger points (MTrPs) (5). Traditionally, MPS has been regarded as a localized issue involving “trigger points” within skeletal muscle (6). In recent years, with the advancement of biomechanical and histological research, the fascial system has come to be regarded as a central contributor to the pathophysiology of MPS (7). The fascial system comprises aponeurotic expansions and epimysial fascia. Aponeurotic expansions transmit kinetic energy between adjacent joints and skeletal muscle groups, their precise directionality corresponding to distinct muscular activity patterns. The epimysium adheres closely to muscles, with its collagen fibers dynamically adjusting orientation according to muscular state. It plays a decisive role in force transmission to skeletal levers and is rich in free nerve endings closely linked to muscle spindles. If the fascia undergoes densification, it impedes normal muscle spindle contraction, inhibits coordinated activation of associated muscle fibers, and leads to movement dysregulation and joint imbalance, thereby inducing pain. Conversely, excessive fascial stretching may sustain muscle spindle excitation, inducing abnormal acetylcholine release at trigger points. Adaptive alterations in connective tissue following trauma constitute the root cause of latent motor dysfunction. This establishes that connective tissue abnormalities within periarticular muscle groups are the fundamental cause of joint imbalance and sensory abnormalities (8–10).

It must be noted that there is a fundamental limitation in the field of MPS: the lack of universally accepted and reliable diagnostic criteria. Although consensus-building efforts based on the Delphi method have been undertaken, diagnosis still relies primarily on subjective clinical examination—particularly palpation of tender points—with limited inter-rater agreement and a lack of validated objective biomarkers (11, 12). This diagnostic uncertainty provides an important backdrop to the mechanistic studies discussed later in this paper. In light of the diagnostic challenges outlined above, this paper adopts the multifactorial pathological model of MPS as its conceptual framework. Within this framework, the fascial system is regarded as one of several contributing factors that interact with muscles, nerves, central sensitization and psychosocial factors, rather than as a single explanatory mechanism (13–15). This integrative perspective underpins the theoretical framework of this paper.

It should be noted that fascial tissue contains a wealth of free nerve endings; this anatomical fact suggests that fascia may be involved in the transmission of sensory signals. However, anatomical plausibility does not equate to a proven clinical causal relationship—the mere presence of nociceptors does not imply that fascia is the primary cause of chronic pain. Current evidence supports a multifactorial pathological model of MPS; fascia should be regarded as one of several contributing factors interacting with muscle dysfunction, central sensitization and psychosocial factors, rather than a single explanatory mechanism (16–19).

## Viewpoint: a hypothetical framework requiring verification

Most treatment recommendations for MPS employ a multimodal approach encompassing patient education, exercise, behavioral modification, pharmacological interventions, and procedural interventions. Commonly prescribed medications include topical analgesics, non-steroidal anti-inflammatory drugs, and muscle relaxants. Procedural interventions encompass manual therapy, dry needling, trigger point injections, acupuncture, kinesiology taping, transcutaneous electrical nerve stimulation, extracorporeal shock wave therapy, and low-level laser therapy (11, 20, 21). Symptom relief is typically achieved when these interventions are applied early in the disease course.

The core hypothesis put forward in this paper is that manual therapy is, in essence, a biomechanical treatment method that applies specific mechanical loads to normalize fascial tension and restore the function of the fascial system. Its mechanism for treating MPS involves not only releasing local muscles and fascia but also regulating an integrated pathway spanning the “mechanics-tissue-cell-nerve-brain” system (22, 23).

Fascia not only provides structural support for muscles but, more significantly, functions as an integrated mechanoreceptor system. Changes in fascial tension directly influence the sensitivity of muscle and tendon spindles, thereby regulating muscle tension states (24). This mechanical dialogue between musculofascial structures underpins normal motor function. Restricted interfascial gliding or abnormal tension disrupts coordinated muscle patterns, precipitating pain and dysfunction.

The clinical efficacy of manual therapy for myofascial pain syndrome is supported by multiple randomized controlled trials and systematic reviews (25–29), though its core biological targets remain debated. Traditional perspectives tend to attribute its effects to muscle tissue relaxation. However, this viewpoint overlooks a critical anatomical structure—the fascial system. As a dense connective tissue network spanning the entire body, fascia not only provides mechanical support but is also rich in sensory nerve endings, serving as a major source of somatic afferents.

Current research paradigms predominantly rely on clinical endpoint measures such as subjective pain scores and pressure pain thresholds. While these confirm intervention efficacy, they fail to elucidate underlying biological mechanisms. A fundamental limitation lies in the widespread absence of objective, quantitative measurements of changes in fascial tissue structure, biomechanical properties, and molecular environment following manual interventions. Notably, from a biomechanical perspective, the complex mechanical loads generated by manual techniques align closely with the mechanical properties of the fascial network. As a continuous tension system, fascia constitutes an ideal structure for perceiving and responding to such mechanical stimuli (30). Systematic reviews indicate that manual techniques effectively reduce pain intensity and improve function in MPS patients. However, a blind spot in understanding mechanisms lies in our default assumption of a linear “manual technique-muscle-therapeutic effect” relationship, failing to explore the more complex pathway of “manual technique-fascia-biomechanical signaling-clinical efficacy.”

*In vitro* cellular studies suggest that manual techniques may induce fibroblast remodeling and regulate the extracellular matrix. Based on these findings, it is hypothesized that mechanical forces mimicking manual techniques may activate integrin-cytoskeletal pathways, potentially prompting morphological changes in fibroblasts and modulating the synthesis-degradation balance of the extracellular matrix, which could in turn enhance the extensibility and elasticity of the fascia (31). Animal model studies suggest that manual techniques may restore the glide and viscoelasticity of fascia. It has been proposed that the shear forces generated by these techniques may effectively break down the three-dimensional superstructure formed by hyaluronic acid aggregates, which could reduce viscous resistance between fascial layers and potentially restore normal interstitial gliding function. At the same time, mechanical loading may enhance tissue viscoelasticity by altering its rheological properties (32, 33). Collectively, these findings are consistent with the hypothesis that manual therapy's mechanical signals are primarily received by the highly sensitive fascial tissue, potentially triggering adaptive alterations from molecular to structural levels. They further suggest that fascia may function not merely as a passive recipient of manual forces, but potentially as an active biological signal transducer.

In recent years, researchers have begun to uncover the molecular basis of fascia acting as a “signal transducer”. At the pathological level of myofascial trigger points (MTrPs), local fibrosis is a key characteristic. Studies have shown that MTrP tissue exhibits increased collagen deposition and heightened tissue stiffness, alongside upregulation of TGF-β1 and p-Smad2/3 expression, which is consistent with the hypothesis that the TGF-β1/Smad2/3 pathway may mediate the fibrotic pathology of MTrPs. Furthermore, mechanotransduction pathways are thought to play a crucial role in tissue repair. Animal studies have found that manual therapy is associated with downregulation of Piezo1 expression and Ca^2+^ influx, whilst upregulation of the nuclear expression of YAP/TAZ, findings that suggest a potential role in promoting Schwann cell proliferation and myelin regeneration; further research has similarly indicated that YAP/TAZ may be key regulatory factors in post-injury myelin regeneration (34–37). Based on this, we propose the hypothesis that the mechanical load of manual therapy may, by inhibiting TGF-β1/Smad2/3-mediated fibrosis and regulating the Piezo1/YAP/TAZ mechanotransduction pathway, collectively constitute a signaling cascade from “mechanical stimulation” to “tissue repair”.

## Clinical heterogeneity and potential phenotypes of MPS

In clinical practice, not all patients with suspected MPS present with clearly identifiable myofascial trigger points; some patients exhibit distinct palpable nodules and referred pain patterns, whilst others primarily present with diffuse tenderness without any distinct nodules. This variability suggests that MPS may not be a single, homogeneous entity, but rather a spectrum of related disorders with different predominant pathophysiological drivers. Against this backdrop, it may be valuable to consider hypothesis-driven phenotypic approaches (18), such as distinguishing between the following types:

Muscle-dominant phenotype: characterized by distinct trigger points and motor end-plate dysfunction

Fascial-dominant phenotype: characterized by fascial stiffness, impaired gliding, or diffuse tenderness, but without distinct trigger points.

This conceptual distinction helps to explain the variability in clinical presentation and treatment response, and provides a framework for future research and personalized treatment strategies. It should be noted that this phenotypic classification is currently only a working hypothesis and requires validation through systematic research.

## Mechanisms of mechanical stimulation in manual therapy: an analytical framework

The primary challenges facing the clinical application of manual techniques lie in their non-standardized nature and unclear mechanisms, coupled with a lack of systematic guidance grounded in neuroscientific principles. These factors collectively limit their widespread adoption and recognition within modern healthcare systems (38, 39). However, advances in contemporary biomechanics offer novel approaches to addressing these limitations, presenting significant opportunities for systematically decoding the “mechanical language” of manual techniques. Based on this, we propose a hypothetical framework that deconstructs common manual techniques into three fundamental mechanical stimulation patterns. It should be noted that this framework serves as an analytical tool to guide research, and the various models often appear in combination in clinical practice.

Deep static pressure primarily applies sustained compressive stress; its proposed mechanisms include the modulation of the spatial distribution of inflammatory mediators and the reduction of nociceptor sensitivity through alterations in local hemodynamics and the cellular metabolic environment, thereby exerting an analgesic effect. At the same time, this intervention may significantly influence fascial fluid dynamics, modulating local immune responses by promoting the flow of interstitial fluid (31, 40–42).

Rhythmic manipulation induces cyclic compression-tension strain, which may effectively stimulate mechanoreceptors within the fascia. Through a gate-control mechanism, this inhibits cytokine expression following nerve injury, thereby alleviating neuropathic pain, whilst simultaneously promoting the proliferation and differentiation of satellite cells and accelerating muscle repair (43–46).

Transverse plucking generates high-intensity shear forces that act directly on abnormal mechanical structures within the myofascial unit, disrupting abnormal collagen cross-links. This stimulation may significantly influence the morphological and gene expression remodeling of fibroblasts, whilst regulating the immunomodulatory mechanisms of fascial tissue (47, 48).

Once these mechanical signals are detected by fascial fibroblasts and immune cells, they may be converted into biochemical signals via force-transduction mechanisms (38, 49). This regulates local inflammatory levels and neurotrophic factor release (50), creating a microenvironment conducive to tissue repair while reducing peripheral nerve sensitivity. Crucially, normalized mechanical information ascends via primary afferent nerves, modulating abnormal reorganizations in the somatosensory cortex and enhancing descending pain inhibitory pathways (34, 51, 52). This pathway provides an integrated biological explanation for the role of manual therapy in the management of chronic pain. This theoretical framework guides modern manual therapy research, which should focus on exploring the regulatory mechanisms of different mechanical stimulation patterns. It aims to establish personalized treatment protocols based on multisystem regulation theory, propelling manual therapy from an empirical technique toward precision medical intervention.

## Future research areas: multimodal research platforms

To validate the aforementioned perspectives and advance manual therapy toward precision medicine, we advocate that future research should center on the following multimodal platforms (53). It should be emphasized that these areas should be regarded as a research agenda rather than established clinical paradigms.

Biomechanical and Imaging Assessment: employ shear wave elastography to quantitatively measure local fascial Young's modulus, thickness, and strain rate before and after manual intervention, objectively documenting dynamic changes in biomechanical properties to enable portable, non-invasive efficacy evaluation (35, 54, 55).

Molecular Microenvironment Monitoring: employ microdialysis techniques to measure real-time concentration changes of bioactive substances—including inflammatory mediators and cytokines—within the interstitial space, revealing manual therapy's dynamic regulatory effects on the local biochemical microenvironment (56–58).

Multi-omics integration analysis: combining transcriptomics and proteomics through micro-tissue biopsy or non-invasive sampling, comprehensively deciphering gene and protein expression profiles in fascial tissue following manual mechanical loading to elucidate molecular mechanism networks at the systems level (59).

Central nervous system response validation: employing high-resolution electroencephalography and functional magnetic resonance imaging to precisely capture how manual techniques modulate functional connectivity and activity within the brain's pain matrix via somatosensory afferents (60, 61). This helps to support the “brain-fascia axis” hypothesis, although it should be noted that this concept remains at the hypothetical stage.

**Figure 1:**
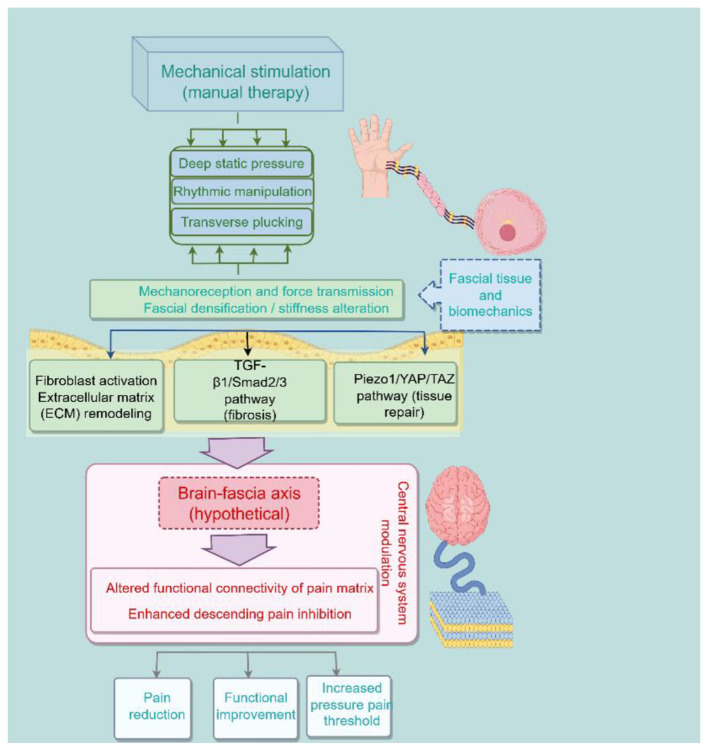
Schematic diagram illustrating the “mechanical-fascial-cellular-brain” axis concept in the manual treatment of myofascial pain syndrome.

Based on this research platform, precise biomechanical parameter systems for diverse manual techniques can be established. This will advance future clinical practice toward “precision biomechanical manipulation”—individualized prescription of biomechanical stimulation patterns (intensity, frequency, direction, duration) tailored to patients' specific fascial phenotypes, achieving a paradigm shift from empirical techniques to precision medical interventions. However, it must be emphasized that this vision is currently still at the conceptual exploration stage, and a great deal of research remains to be done before it can be applied clinically. To visually summarize the proposed “mechanics-fascia-cell-brain” axis, a conceptual diagram is provided in Figure 1.

Caption: This figure summarizes the hypothetical framework proposed in this paper. The left panel shows the three fundamental mechanical stimulation patterns of manual therapy: deep static pressure, rhythmic manipulation, and transverse plucking. These mechanical forces act on the fascial tissue and are perceived and transmitted by the mechanoreceptors within the fascial system (fascial densification/stiffness alteration). Subsequently, the mechanical signals are transduced into cellular and molecular responses, including fibroblast activation, extracellular matrix (ECM) remodeling, the TGF-β1/Smad2/3 pathway (associated with fibrosis), and the Piezo1/YAP/TAZ pathway (associated with tissue repair). Normalized afferent signals then modulate central nervous system activity via the hypothetical “brain-fascia axis”, leading to altered functional connectivity of the pain matrix and enhanced descending pain inhibition. The framework culminates in clinical outcomes: pain reduction, functional improvement, and increased pressure pain threshold. The dashed box (“brain-fascia axis”) indicates a hypothetical construct that requires human validation. Solid arrows represent pathways supported by preclinical or *in vitro* evidence.

## Conclusion

We propose a novel conceptual framework: manual therapy can be regarded as a precise biomechanical intervention targeting fascial structures, treating myofascial pain by modulating peripheral tissue homeostasis and central nervous system networks. Adopting this paradigm shift will pioneer a new pathway for hundreds of millions of MPS patients worldwide, transitioning from empirical treatments toward evidence-based, precise, and personalized non-pharmacological interventions. This paper adopts the multifactorial, biopsychosocial model of MPS as its conceptual framework, viewing the fascial system as one of several contributing factors that interact with muscular dysfunction, central sensitization and psychosocial factors, rather than as a single explanatory mechanism. This integrative perspective underpins the theoretical framework of this paper, aiming to provide a conceptually balanced framework for understanding the biological basis of manual therapy. The ultimate goal is to gradually realize the clinical vision of “precision biomechanical manual therapy” following thorough validation—a vision that should currently be understood as a future research direction rather than an established clinical paradigm.
